# Daily life changes and adaptations investigated in 154 families with a child suffering from a rare disability at a public centre for rare diseases in Northern Italy

**DOI:** 10.1186/s13052-016-0285-0

**Published:** 2016-08-31

**Authors:** G. Silibello, P. Vizziello, M. Gallucci, A. Selicorni, D. Milani, P. F. Ajmone, C. Rigamonti, S. De Stefano, M. F. Bedeschi, Faustina Lalatta

**Affiliations:** 1 Child and Adolescent Neuropsychiatric Unit, Fondazione IRCCS Ca’ Granda Ospedale Maggiore Policlinico, Milan, Italy; 2 Department of Psychology, University of Milano, Bicocca, Italy; 3 Pediatric Genetic Unit, Department of Pediatrics, MBBM Foundation, A.O S Gerardo, Monza, Italy; 4 Pediatric Highly Intensive Care Unit Department of Pathophysiology and Transplantation Fondazione IRCCS Ca’ Granda Ospedale Maggiore Policlinico, Milan, Italy; 5 Clinical Genetics Unit, Fondazione IRCCS Cà Granda Ospedale Maggiore Policlinico, Via Commenda 12, 20122 Milan, Italy

**Keywords:** Rare diseases, Diagnosis, Parental adaptation, Child disability, Lifestyle changes, Public health system

## Abstract

**Background:**

Living with a disabled child has profound effects on the entire family. With a prevalence of developmental disabilities around 2,5 %, there is a considerable need to promote improvements in the health care system. Little is known about changes and adaptations in the lives of affected families and this paucity of information hinders the improvement of services. This study sought to explore the needs and changes in the everyday life of families with children suffering from rare diseases of varying severity, with and without mental disability. The aim was to measure the socio-demographic characteristics, health care problems and living conditions of a large cohort of families with an affected member.

**Methods:**

A sample of 154 families was recruited between September 2011 and April 2013 to respond to a 136 item questionnaire that explored different areas of concern (diagnosis and follow-up of clinical specialists, relationship with pediatrician, rehabilitation, school, work, institutional and/or private support, child care needs and family relationships).

**Results:**

All parents answered the questionnaire. They were satisfied with the services provided in particular for diagnosis and follow-up, relationships with the family pediatrician, rehabilitation services and school, regardless of the severity of condition, presence of intellectual disability (ID) or absence of diagnosis. Negative scores were reported for institutional and/or private support and family relationships in severe conditions.

**Conclusions:**

The Health Care System should maintain a family-centered care and a multi-agency working, improving quality of life of families with disabled child to allow adaptation. At present these services are uncoordinated and financial support is poor, resulting in a heavy burden for these families.

## Background

According to the most recent report from the Italian National Institute of Statistics [1] the prevalence of adults with a disability in Italy is approximately 4.8 % of the general population. The prevalence of a disability occurring during the developmental period is more difficult to estimate. A study by Fondazione Zancan suggests a value of about 2.5 % of children with disabilities [2]. The Italian Health Service is currently facing an enormous challenge to provide high quality services for a population that requires both immediate and continuous care. Unfortunately, there are a number of limiting factors in Italy for people with disability as for example heterogeneity of services, with more resources in the northern part of the country than elsewhere. Financial support for people with disabilities are quite inadequate everywhere, however, resulting in a heavy burden for families which inevitably reduces their quality of life [3]. Specific laws have been established over the last two decades to protect the child and its family but resources have now been reduced, particularly financial and educational support. For example, Law 104/92 sets out the principles of the system with respect to the rights, social integration and support of handicapped persons and regulations allowing for the reduction of a mother’s working hours, according to child’s diagnosis.

In our country, research on the disruptive consequences of one family member’s disability on personal stability and familiar relationships [4] has been poorly developed. There are a large number of non-Italian reports on this subject. For example there is considerable research regarding children with health care special needs (CSHCN) and Intellectual disabilities (ID) and the quality of health care [5–11] that has found that health check programs for adults with ID identify unmet health needs [12, 13]. There are many studies focused on the socioemotional needs of children with chronic medical conditions, like disease-related stress, frustration with medication management, social isolation from peers, and despair at the awareness of limitation and difference from others [14–17]. In addition, care for a child with a chronic condition can be emotionally taxing on family members. Caregiver burden and strain on family financial and emotional resources may increase risk of psychological or family-function problems for many family members [15–18]. Potential family impact includes depression, adjustment problems, and “spillover effects” to siblings [14]. Given these needs, it is important to know whether perceived mental health needs are unmet for children with chronic conditions and for their close family members.

According to Pelentsov et al. [19] parents caring for a child with a rare disease report unmet needs, the origins of which are varied and complex. Few studies have systematically attempted to identify comprehensively the supportive care needs of parents with a child with a rare disease. The authors used the widely accepted Supportive Care Needs Framework (SCNF) which consists of seven domains of supportive care needs like practical, spiritual, social, psychological, informational, emotional and physical needs. In their review they identified the most common areas for these families as social needs, followed by informational and emotional concerns.

In addition to the pathological impact of a disability on family members, the ways in which families cope needs to be analyzed with a focus on personal adjustments, resilience, special needs and support. Zanobini et al. [4], by evaluating correlations between social and demographic aspects including age, education, type of work using a scale of adjustment and coping have demonstrated that most families with a disabled child have the resources to adapt to changes produced by the disability. According to Drotar [20] parental and family adjustments after the birth of a disabled child have a primary influence on the child’s psychological functioning and that less adaptive family relationships (e.g., greater conflict and maternal psychological distress) consistently predicte poorer outcomes for the affected child. Variables which have the greatest impact on family function include: a. the child's situation, specifically, nature and severity of disability, impediments or physical and psychological disorders; b. personal characteristics of the parents (emotional and coping skills); c. family network (family size, quantity and quality of relationships); d. social support in terms of available community resources and services. However, the families of children with rare genetic syndromes have been the focus of surprisingly few research studies [21]. That is, most family research in this area has focused on parents of children with more common conditions associated with ID, such as autism and Down syndrome [22–24]. Griffith et al. [21], did focus on three rare genetic syndromes associated with characteristic behavioural phenotypes: Angelman, Cornelia de Lange and Cri du Chat syndromes. Parents of children with these disabilities and a matched comparison group of parents of children with autism and intellectual disabilities completed questionnaires on both psychological distress (stress, anxiety, depression) and positive psychological functioning. The authors demonstrated that parents of syndromic children were more likely to report clinical levels of negative outcomes, such as anxiety and depression. Horridge et al. [23] had tried to quantify the multifaceted needs of disabled children and their families in Sunderland, north-east England from structured electronic clinical reports of children attending paediatric disability clinics. The required number of clinic appointments correlated strongly with the number of needs identified. The authors concluded that profiling the multifaceted needs of disabled children may prompt more proactive care. Furthermore, earlier identification of known associated conditions and issues, and more timely interventions and advocacy for families, with a mechanism in place to monitor and report outcomes and available support will allow more relevant service and guide care pathway design. Sufficient evidence was provided to economically justify the appointment of additional paediatric disability consultants. Counting numbers of needs and issues quantifies complexity in a straightforward way.

Objectives of our study are to describe the socio-demographic characteristics, health problems and living conditions of a large cohort of families with a child suffering from a rare genetic disease of varying severity, to explore the needs and changes in the everyday lives of these families with the aim to improve our understanding of the quality of services offered to these families in Italy and so as to better support their special needs. We wanted also to better understand how the family adapts to a disabled child by examining such variables as age at diagnosis, follow-up services, role of the pediatrician and clinical specialists, rehabilitation services, school, work, institutional and/or private support, child care needs and family relationships using parental interviews, to collect data on the quality of services offered by the established Italian welfare agencies and parental satisfaction and their difficulties in coping with everyday life situations.

## Methods

Patients were recruited from children affected by a rare disease, referred to the Pediatric Genetic Units, Clinical Genetics Unit, or Neuropsychiatry Unit (Unit of Complex Disability and Rare diseases) at Fondazione IRCCS Cà Granda Maggiore Policlinico Hospital of Milan, a large tertiary Centre in northern Italy. This Institution hosts a number of highly specialized services which deal with rare diseases and functions as a reference center for the entire geographic region. The sample is representative of all the affected families, being that it is clinically heterogeneous and cuts across societal and medical boundaries.

The parents of our sample of 154 patients were invited to take part to the study at the end of a clinical evaluation of their child during a routine follow-up. Questionnaires were given to parents between September 2011 and April 2013. There were 72 girls and 79 boys (3 children had a missing indication for gender) with an average age of 7.04 years (SD = 4.69). Table 1 lists the clinical and genetic diagnosis of the probands.

**Table 1:** Clinical and genetic diagnosis of our sample

Diagnosis	Number	Percent
Without specific diagnosis	20	12,9
Neurofibromatosis type 1	13	8,4
Williams syndrome	12	7,8
Hemihypertrophy	11	7,1
Cornelia De Lange syndrome	8	5,2
Achondroplasia	7	4,5
Microdeletion 22q11.12	5	3,2
Down syndrome	4	2,6
Rubinstein-Taybi syndrome	4	2,6
Hypochondroplasia	3	1,9
Syndrome unknown	3	2,9
Charge syndrome	2	1,3
Gorlin syndrome	2	1,3
Kabuki syndrome	2	1,3
Leopard syndrome	2	1,3
Noonan syndrome	2	1,3
Poland syndrome	2	1,3
Proximal spinal muscular atrophy	2	1,3
Psychomotor retardation	2	1,3
Silver Russel syndrome	2	1,3
Aarskog syndrome Angelman syndrome Autism Bardet Biedl syndrome Cardiofaciocutaneous syndrome Cerebral ventriculomegaly and aortic coarctation Citrullinemia Cockayne syndrome Cohen syndrome Coloboma of iris associated with gastro-intestinal reflux and unilateral deafness Congenital encephalopathy Costello syndrome Crouzon syndrome Cutis laxa Deafness Deletion 13p Deletion 18q Deletion 6p Duchenne muscular dystrophy Fanconi Anemia Fetopathy alcoholic Freeman Sheldon syndrome Frontofacial syndrome Goldenhar syndrome Hallermann Streiff syndrome Holt Oram syndrome Kniest dysplasia Lujan Fryns syndrome Marden Walker syndrome Mosaic variegated aneuploidy Mowat Wilson syndrome Mucopolysaccharidosis Paraparesis ataxic Pontocerebellar hypoplasia type 2 Prader Willi syndrome Smith-Magenis syndrome Sotos syndrome Stickler syndrome Syndrome polymalformative unknown Treacher Collins syndrome Trisomy X (47,XXX) Tsukahara syndrome Tuberous sclerosis Ulnar-mammary syndrome Unspecific chromosome alteration Wiskott Aldrich syndrome	1	0,6

We used a modified version of a Questionnaire [4] that been used by Zanobini et al. previously, to assess different aspects of family life and, in particular, to investigate the perceived quality of the health care services. This Questionnaire has not been previously validated but was built for the express purpose of obtaining a qualitative data collection regarding the ability of families with a disabled child to adjust to the necessities of raisng such a child. The format includes the following areas using multiple choice questions, open questions and Likert scales:

1. *Institutional Support received at time of diagnosis and follow-up*. This includes relationships among family and pediatrician and other clinical specialists, rehabilitation services, the child’s school, parent’s workplace, and institutional and/or private support.
2. *Child care needs and Family Relationship*.
3. *Scale of Personal Adjustment*.
4. *Brief-COPE*. This assessed the personal reactions of the parents in relation to the commitment required by the illness of the child. The Brief-COPE, first ideated by Charles S. Carver [25], is a reduced version of the COPE Inventory of Carver, Sheier and Weintraub [26].
5. *Personal Data* were also collected including family composition, demographic data and socio-cultural aspects of the different members of the family.

The research project was submitted to the Ethical Committee for formal approval but the need for approval was waived, according to the actual standards. After written consent the parents of the enrolled children were asked to fill in an anonymous 136 item questionnaire. The collected data were entered into a database built in collaboration with the Institute of Statistics and Informatics, University of Milano-Bicocca. Frequency data were tested using Chi-square tests, whereas continuous variables associations were analyzed by Pearson correlation and, when necessary, analysis of variance.

## Results

Table 2 summarizes the demographic data of our population.

**Table 2:** Demographic data

Demografic data											
Respondent			Patient sex			Patient age			Parents age		
	*N*	*%*		*N*	*%*		*M*	*SD*		*M*	*SD*
Mother	108	70.1	Boys	79	51,3	Patient age	7,04	4,69	Father	42,38	7,19
Father	46	29.9	Girls	73	47,4				Mother	39,08	6,6
* total*	154	100	*total*	152	98,7						
Fathers' study			Mothers' study			Fathers' work			Mothers' work		
	*N*	*%*		*N*	*%*		*N*	*%*		*N*	*%*
High school	47	30,5	High school	56	36,4	Employee	40	26	Employee	51	30,6
Middle school	45	29,2	Middle school	34	22,1	Freelance	31	20	Housewife	46	29,9
Degree	28	18,2	Degree	32	20,8	Specialized workman	24	15,6	Teacher	11	7,1
3-year diploma	19	12,3	3-year diploma	28	18,1	Artisan	17	11	Freelance	9	5,8
Primary school	7	4,5	Primary school	2	1,3	Manager	9	5,8	Specialized workman	8	5,2
* total*	146	94,8	*total*	152	98,7	No-specialized workman	9	5,8	No-specialized workman	8	5,2
						Merchant	5	3,2	Other works	5	3,2
Parents conjugality						Other works	5	3,2	Pensioner	5	3,2
	*N*	*%*				Unemployed	2	1,3	Merchant	3	1,9
Married	117	76				Soldier	2	1,2	Manager	2	1,3
Unmarried couple	28	18,2				Pensioner	1	0,6	Artisan	1	0,6
Divorced	6	3,9				*total*	154	100	*total*	154	100
Widowed	1	0,6									
* total*	152	98,7									

The children’s medical conditions were divided into three categories according to the clinical expression of their condition: 13.0 % (*N* = 20) of the cases were considered to be mildly affected, 50 % (N = 77) were of medium severity while 37.0 % (*N* = 57) had a severe condition. Table 3 describes the parameters used to define the three groups. It is important to note that the severity groups and classification of ID were based on the average clinical and genetic characteristics of the syndrome affecting the child. This classification was initiated by a clinical geneticist with extensive experience with rare diseases and with the advice and superviion of the multidisciplinary group. Severity groups were balanced across gender (*X*^2^ = 1.400, *p*. = .497). However, when groups are compared according to the parents perception of the severity of the child's condition (with a score of 0 being very mild and 4 very severe), groups differed, because children classified as severe by the medical professionals scored higher (less severe) (*M* = 2.15) than medium (*M* = 1.85) and mild (M = 1.35) groups (F(2,147) = 3.627, *p*. = .029).

**Table 3:** Severity of diagnosis in our sample

Severity of diagnosis		
Mild	Medium	Severe
localized and limited esthetics	• unlocalized • costitutional • no mental retardation • health charging necessary and ongoing	• multiple malformation • costitutional • mental retardation • health charging necessary and ongoing
Aarskog syndrome; Cutis laxa; EmiperHemihypertrophytrofia; Holt Oram syndrome; Poland syndrome; Silver Russel syndrome; Trisomy X (47,XXX); Ulnar-mammary syndrome.	Achondroplasia; Bardet Biedl syndrome; Cerebral ventriculomegaly and aortic coarctation; Charge syndrome; Citrullinemia; Coloboma of iris associated with gastro-intestinal reflux and unilateral deafness; Crouzon syndrome; Deafness; Freeman Sheldon syndrome; Goldenhar syndrome; Gorlin syndrome; Hypochondroplasia; Kniest dysplasia; Leopard syndrome; Microdeletion 22q11.12; Mosaic variegated aneuploidy; Neurofibromatosis type 1; No diagnosis; Noonan syndrome; Paraparesis ataxic; Psychomotor retardation; Stickler syndrome; Syndrome polymalformative unknown; Treacher Collins syndrome; Tsukahara syndrome; Tuberous sclerosis; Wiskott Aldrich syndrome.	Angelman syndrome; Autism; Cardiofaciocutaneous syndrome; Cockayne syndrome; Cohen syndrome; Congenital encephalopathy; Cornelia De Lange syndrome; Costello syndrome; Deletion 13p; Deletion 18q; Deletion 6p; Down syndrome; Duchenne muscular dystrophy; Fanconi Anemia; Fetopathy alcoholic; Frontofacial syndrome; Hallermann Streiff syndrome; Hallermann Streiff syndrome; Kabuki syndrome; Lujan Fryns syndrome; Marden Walker syndrome; Mowat Wilson syndrome; Mucopolysaccharidosis; Pontocerebellar hypoplasia type 2; Prader Willi syndrome; Proximal spinal muscular atrophy; Rubinstein-Taybi syndrome; Smith-Magenis syndrome; Sotos syndrome; Unspecific chromosome alteration; Williams syndrome.

Because we were interested in the relevance of intellectual disability (ID) to the patients' lives we also divided the patients into three ID categories with 57 (37 %) of patients classified as without, 38 (24.7 %) with mild, and 59 (38.3 %) with severe ID.

Finally, we also investigated the effects of having received a diagnosis (125 patients) compared to not having received one (29 patients).

Results from each subsection of the questionnaire:

### Institutional Support received over time

Taking into account mode of diagnosis and control services by the clinical specialist, children received their first diagnosis within their third year of life (mean = 2.6, SD = 3.6), most frequently by their pediatrician (46.8 %, N = 72) and less frequently by the gynecologist (13.6 %, *N* = 21) or geneticist (7.1 %, *N* = 11). The degree of severity did not affect the age at diagnosis (F(2,129) = 1.308, *p*. = .274), nor the source of the diagnosis, or presence of ID (F(2,129) = .535, *p*. = .587). Children underwent medical checks on a regular basis, mostly at the day hospital (62 %) or while visiting a specialist (42 %), with a few children requiring hospitalization (13 %). Follow-ups (*X*^2^(2) = .877, *p*. = .645) and day hospital admissions (*X*^2^(2) = .514, *p*. = .773) were equally frequent in the severity classes, averaging at 46 % and 60.7 % in the sample, and for the presence or absence of ID (follow-ups *X*^2^(2) = .311, *p*. = .856, hospital admission *X*^2^(2) = 2.775, *p*. = .250. Specialist examinations were less frequent (*X*^2^(2) = 7.627, *p*. = .022) in mild conditions (15.0 %) than in all other groups (medium = 45.2 %, severe = 50.0 %). Hospitalization was infrequent (12 % for the entire sample), with no difference across severity groups, degree of ID or presence of a diagnosis. It is important to note that we did not have information about how the family lived before the diagnosis.

About 90 % of the sample received a clinical evaluation by the pediatrician once a month (37.7 %) or once every six months (32.5 %). The remaining 19.8 % were evaluated one a year. Examinations usually required less than an hour (87 % of cases), with only 16 cases reporting longer visits of up to 4 h. The severity groups did not differ in the frequency or the length of the visit to the pediatrician.

As regards, instead, rehabilitation services forty-four percent of the children in our sample currently undergo physiotherapy, 40 % psychomotor rehabilitation and 42 % speech therapy. For 72 % of cases the rehabilitation sessions were held at home with the remainder at the specialist’s office or at the hospital. On average, rehabilitation requires 1.5 h weekly. Rehabilitation programs where not different across severity, ID or presence or absence of diagnosis groups.

Children attended school according to their regular national and regional schedules. Children were brought to school generally by the parents (61 %) or other family members (2.6 %), with extra help required only by 4.5 % of the families. Fifty-five percent of children needed support in the classroom either by a dedicated teacher or a communication assistant from the public service. The children’s school performance on personalized programs, as reported by parents, was generally good: Only 3.5 % of children showed an insufficient level of performance; 30.7 % of parents reported their children performance as being “good enough”, 42.1 % as “good”, and 23.7 % as “excellent”. Performance, however, was strongly influenced by the severity of the medical condition *X*^2^(6) = 23.307, *p*. = 001: mild and medium severity groups averaged between “good enough” and “good”, severe conditions groups averaged between “insufficient” and “good enough”. The four children ranked as “insufficient” were in the severely affected group. Children with ID generally performed less well at school (averaging between “insufficient” and “sufficient”) than did children with normal mental skills (F(2,111) = 6.138, *p*. = .003). The presence or absence of a diagnosis did not affect school performance.

In our country, similar to many European countries, families having children with disabilities may take advantage of a series of support programs. In our sample, 48 % of the families received support from social services, 41 % from non-profit organizations, and 20 % from the day care service by the Health Care and/or Social System. Families also received help from relatives (27 %) and friends (65 %). No difference was observed according to severity, ID or the presence or absence of a diagnosis.

All families received financial help from the state, and parents did benefit from paid leave from work (66 %) and other benefits.

Interestingly, families were generally informed about the availability of financial help and social services dedicated to disabilities primarily by the medical specialists (61 %) or their general practitioner (21 %). Only a minority (6 %) were apprised by school personnel or non-profit organizations. Forty percent of parents reported that they got information about their rights and services availability on their own.

Each section of the questionnaire ended with an open question that required the parents to think about the service, asking them what improvements they would like to see in clinical services and social support. Seventy-two caregivers answered as follows: reduced waiting lists, both for booking a visit and for the time spent waiting for the visit at the Hospital (40 % of subjects), better relationships and communication between doctor and patient (25 % of subjects) and improved integration of services and collaboration among specialists (35 %). Thirty caregivers asked for more concrete support on a daily basis and for psychological and emotional help.

Results regarding satisfaction in these institutional supports are reported in Table 4. Families expressed satisfaction with the overall service received during the periodic checks and day hospital admissions. None of the parents' evaluations were different across severity groups, or for the presence or absence of a diagnosis. Satisfaction with the service was different depending on the severity of ID: parents of patients having ID were less satisfied with the problem solving skills offered by the service and found the hospital staff less co-operative than parents of patients without ID (Table 5). Parents' evaluations of relationship with the pediatrician did not differ across severity or ID groups. Parents who did not receive a diagnosis were generally less satisfied (Table 6) with the pediatrician regarding their communication (F(1,138) = 4.67, *p*. = .032) and cooperation (F(1,138) = 6.25, *p*. = .014). Evaluations of the rehabilitation services were generally positive (Table 4). None of the parents' evaluations were different across severity, ID and presence or absence of diagnosis groups. School evaluation by the parents was good (Table 4), independent of severity, ID, and presence of a diagnosis. Not surprisingly, the satisfaction with the school expressed by parents was correlated with the reported performance of the child: interaction with the personnel (r = .277, *p*. =004), quality of teachers (*r* = .313, *p*. = 001), didactic competence of teachers (*r* = .250, *p*. = 011), didactic competence of supporting teacher and personnel (*r* = .219, *p*. = 085), and cooperation (*r* = .280, *p*. = 004) were all positively related to the child’s performance. When asked to evaluate the procedures needed to obtain financial support (in terms of paperwork and delays), families were generally unhappy about the service (average 1.6 where 1 is very unsatisfied and 4 very satisfied).

**Table 4:** Parents satisfaction to Institutional Support received over time

Satisfaction to institutional support^c^								
	Relationship		Communication^a^		Problem-solving^b^		Cooperation	
	M	SD	M	SD	M	SD	M	SD
Diagnosis and Control Services of the Clinical Specialist	3.34	.701	3.34	.763	3.32	.839	3.33	.839
Pediatrician	2.92	.964	2.99	.926	2.78	1.080	3.01	.962
Rehabilitation Services	3.14	.954	3.15	.893	3.15	.949	3.25	.903
School	2.94	.998	2.95	1.016	2.90	.995	3.25	.847

^a^quality of the information received

^b^manner in which problems were handled by

^c^scale from 0 (very unsatisfied) to 4 (very satisfied)

**Table 5:** Parents' satisfaction with the hospital service by severity of patient ID

Intellectual Disability (ID)					
	Absent	Mild	Severe	F	*p*
Communication	3.52	3.18	3.26	2.70	*.070*
Problem solving	3.56	3.14	3.14	3.68	*.028*
Cooperation	3.60	3.19	3.16	4.86	*.009*

**Table 6:** Parents' satisfaction with the pediatrician broken down by presence of a diagnosis

Specific diagnosis received				
	No	Yes	F	*p*
Communication	2.63	3.07	4.67	*.032*
Problem solving	2.50	2.83	1.76	*.186*
Cooperation	2.57	3.11	6.26	*.014*

### Child care needs and family relationship

Analyzing child care needs in questionnaire, we estimated the family burden in assisting a child with disabilities during their daily activities was as the time needed to carry out the essential daily routines and the sharing of the child support activities within the family. The majority of parents reported that taking care of the child’s personal hygiene required less than (42 % of cases), or about an hour a day (38.1 %). Similar times were reported for eating. The time required to fall asleep was generally within one hour (95 % of cases). No statistical difference was observed across severity groups, ID, and presence/absence of diagnosis.

A large proportion of parents (83 %) reported that the support activities were shared within the family, with 60 % asserting that the activities were shared very often between mother and father. A few families reported sharing the support of daily activities with other members of the family (6 %), friends (2 %), or paid help from housekeepers (3 %). No difference appeared across different groups of patients.

When asked about their perception of the burden of assisting the child in daily activities, parents reported their effort as not particularly heavy (mean 2.06 in a scale from 0, not heavy at all, to 4 very heavy), although they generally worried about the future needs of their children (mean 3.21 in a scale from 0, not worried at all, to 4 very worried). Interestingly, no difference was observed across severity groups or presence/absence of diagnosis either in terms of perceived difficulties, or future concerns. However, parents with children affected by ID were more worried about the future (mild = 3.35 and severe = 3.39) than parents with children without ID (2.93), F(2,115) = 3.298, *p*. = .040.

The emotional component of coping with the child’s disabilities was investigated by asking parents to report how often different kinds of emotions were expressed in the family (with a scale from 0, never expressing emotion, to 4, very often). Emotional expression was not particularly negative; as happiness and compassion were the most expressed emotions (mean 1.78 and 1.69, respectively). Loneliness (mean = .48), aggression (mean = .58), guilt (mean = .71), and sadness (mean = .98) were less frequent than positive emotions. Anger was reported as expressed “sometimes” (mean = 1.04). Only sadness was more frequent in families with severe conditions (1.07) respect to mild (.72), medium (.98) severity groups (F(2,126) = 4.665,*p*. = .011). Other emotions were reported at the same levels across the severity groups. Emotional expression was not related to the age of the patient - with r ranging from -.079 (*p*. = .359) for happiness to .150 (*p*. = .090) for loneliness nor with ID, with r’s from -.018 (*p*. = .837) for happiness to .144 (*p*. = .098) for anger. Having or not having received a diagnosis or the presence or absence of ID did not influence emotional expression.

### Scale of personal adjustment

Parents were asked to report whether the child’s disability had generated changes that they considered beneficial (mean response greater than 2) or detrimental (mean response less then 2) for their quality of life, the scale being that a response of 2.0 indicated no significant change. Negative changes were reported in the financial situation (mean = 1.66, SD = .662) and work status (mean = 1.55, SD = .740). The emotional experience was reported as changed toward a more negative status (mean = 1.69, SD = .849). No important changes were reported for the relationship with their partner (mean = 2.02, SD = .666), relatives (mean = 1.97, SD = .582) or friends (mean = 1.92, SD = .560).

The severity of the condition seemed to be associated with the presence of work-related changes and changes to the couple’s relationship. Interestingly, severe conditions are more associated with negative consequences of work-related changes, F (2,139) = 4.147, *p*. = .018, and economic changes F(2,140) = 4..214 *p*. = .017, than with other life changes. The presence of ID had a strong effect on the parent’s stability (Table 7). Parents with children with ID (mild or severe) reported more negative consequences arising from work-related problems, housing, financial situation and relationships with the partner and relatives.

**Table 7:** Consequences of having a child with intellectual disabilities (ID) on the parent’s lives (lower values indicate negative changes, with 2.0 being unchanged)

Intellectual Disability (ID)				
Category	Absent	Mild	Severe	F,p
Work status	1.76	1.63	1.30	5.82, 0.004
Housing	2.04	1.91	1.84	2.82, 0.063
Financial	1.92	1.56	1.47	7.36, 0.001
Partner	2.24	1.94	1.88	4.40, 0.014
Relatives	2.13	1.97	1.81	4.43, 0.014
Others	2.21	2.09	1.82	4.24, 0.016

The way parents cope with different life changes is associated strongly with the emotional expression reported by the parents. In particular, parents who reported more frequent expression of happiness in the family were also more likely to cope positively with work-relate changes (*r* = .227, *p*. = .008), changes in the couple’s relationship (*r* = .289, *p*. = .001), in relationships with relatives (*r* = .296, *p*. = .001), friends (*r* = .175, *p*. = .041), and changes in the emotional status (r = .221, *p*. = .010). Conversely, loneliness was negatively associated with coping with life changes: parents who reported more frequent expressions of loneliness in the family were also more likely to cope negatively with work-relate changes (*r* = −.297, *p*. = .008), changes in the couple (*r* = −.321, *p*. < .001), relationships with co-workers (*r* = −.201, *p*. = .025), friends (*r* = −.211, *p*. = .018), and in the financial situation (*r* = −.321, *p*. < .001).

Coping with life changes was also associated with concerns about the child’s future: in particular, worries were associated negatively with positive changes in the work-related (*r* = −227, *p*. =.003) and positive financial changes (r = −.329, *p*. <.001).

### Coping

Significant correlations between coping and socio-cultural aspects were uncovered by analyzing the Brief-COPE questionnaire.

The child gender seems to be correlated with “Positive Reframing” but there is a positive correlation only with respect to the mother (*r* = 0.204, *p* < 0.05). The age of the child with disability correlated to the strategy of “Acceptance” (*r* = −0.210, *p* < 0.05). The educational level of the parents, in contrast, seems to correlated with additional aspects of coping: “Behavioral Disengagement” correlated negatively both in mother (*r* = − 0.194, *p* <0.05) and father (*r* = −0,185, *p* <0.05) to the level of education while “Self-Distraction” correlated positively in the mother (*r* = 0.184, *p* < 0.05); “Positive Reframing” correlated negatively in father (*r* = −0.219, *p* < 0.05). The numbers of children correlated positively with Self-Distraction (*r* = 0.236, *p* < 0.05), “Denial” (*r* = 0.176, *p* < 0.05) and “Self Blame” (*r* = 0.173, *p* < 0.05). The strategy of “Planning” correlated positively with the marital status of the couple (*r* = 0.189, *p* < 0.05).

## Discussion

One of the aims of the study was to investigate the relationships between a family with a disabled child and the “Specialized Services” and how parents become an integral part of the care team of their child (Family Centered Care). It is well accepted that access to health care is still critical for people with disabilities in Italy.

Few studies have investigated the needs and rights of inhomogeneous populations lacking a diagnosis although several studies exist concerning well-defined and homogeneous population such as Down syndrome [27–31], Williams syndrome, Fragile X syndrome, Autism [32, 33], Angelman, Cornelia de Lange [34] and Cri du Chat syndrome [21].

The hypothesis that the clinical or genetic diagnosis of the condition underlying the disability is essential for good cooperation among the involved parties is not confirmed by our study and the very satisfactory relationships found in our study group are probably due to the high profile professionals at the reference centers who are able to guarantee care and good communication despite the presence or absence of evidence for a clear etiology. Nevertheless, in contrast with the results reported by Zanobini et al. [4], our families were well satisfied with the diagnostic services and by the communication style even though almost 19 % of our patients were undiagnosed at the time of study. This is probably related to the attitude of the multidisciplinary team to deal with disability and the strong clinical expertise showed by the professionals. That is, caregivers were able to provide all of the information required to meet those needs indentified by Pelentsov et al. [19].

In general, all families in our sample were satisfied with the medical services (hospital admission and outpatient visits), except in conditions involving ID. This may be related to the severity of the condition per se.

All families seem satisfied with the rehabilitation services but 72 % of children benefited from home rehabilitation after parental training. We do not know if rehabilitation at home is a choice or a necessity brought about by either a complete lack of public services or very long waiting lists. According to Zanobini et al. [4], parents feel themselves to be active in care and are relieved when they are able to rehabilitate their child at home. At the time of the study all children went to a mainstream public school. In 55 % of cases children had a requirement for special educational needs. As expected, parents who have children with a severe disability or ID are less satisfied than otherfamilies. Some of the parents also have the burden of taking the child to school probably because of the poor support of other family members and lack of specialized public transport. Due to the fact that school performance correlates with parental satisfaction, it is possible that parents of severely affected children expected less from them and these children might have performed better had they been supported more.

Each parent takes more than an hour to dress, feed and put their child to sleep, regardless of the child’s age. Most parents (83 %) cannot delegate these tasks to others. This could be due to a segregation of the family and difficulties in sharing the problem outside the immediate family.

As expected, only sadness was more frequent in families with severe conditions, while other emotions were reported at about the same levels across the severity groups, according to Griffiths et al. [21]

The severity of condition and the presence of ID were associated with more negative changes in the couple. Our data are comparable with Zanobini et al. [4]. Brickman and Campbell [35] argued that people have a natural tendency to adapt to new situations, even though adverse, with a positive mood. In our sample this mood is present only in people that have a tendency to express positive emotions.

“Behavioral Disengagement” is considered an adaptive strategy to cope with stress [36]: a high level of education increases awareness of the disease and reduces the tendency to focus only on negative feelings connected to the disability. The presence of other children in the family compels parents to activate a particular kind of attitude and the defenses of “Self-Distraction” and “Criticism and Denial”. The negative correlation between the acceptance of the disease and the age of the child is consistent with published studies of families having members with disabilities: the first feelings that appear in the parents after the discovery of the diagnosis are disbelief and “Denial”. At a later stages come the understanding and acceptance of the disease [4, 37].

## Conclusions

Members of our study sample expressed satisfaction with the available Public Health services, the competence of the clinicians and their ability to establish a relationship. Our System should invest more in operator training and in the promotion of Family-Centered Care. Managing the network of sanitary and social services is very complicated for these families. The presence of a dedicated case-manager would seem to be crucial in supplying help and a sense of coherence and continuity to the family. Multiprofessional networking has brought very positive and significant changes for families and children with respect to quality of life, equal access to education and support for health care needs.

This study is limited mainly by the need to construct an ad hoc severity scale (but based on well founded clinical and scientific criteria) as well as the lack of a control group.

Further studies are needed to better focus on the emotional and social needs of families with a disabled child. In particular a better knowledge of the quality of life and un-met needs of families with a disabled child is needed to increase awareness of National Health System providers and promote interventions. Quantitative studies should be conducted on various cohorts of patients and their families during the transitional period. This will allow for the collection of specific data about how families can manage the care of their children regardless of the specific diagnosis. In particular, efforts should be dedicated to:

- continuing medical education (CME) programs concerning the needs of young adults with rare conditions, with or without developmental and intellectual disabilities. This probably whould improve communication among care providers and families with a disabled child;
- education and training of healthcare professionals from all disciplines, including paramedical specialists and non-healthcare professionals such as school teachers and caregivers
- questionnaires asking patients and their families to evaluate their degree of satisfaction, including the areas of the Supportive Care Needs Framework (SCNF), such as practical, spiritual, social, psychological, informational, and emotional and physical needs for improvement of corrective measures
- governments should invest more in increasing the knowledge about disability in Italy, if they do not want to face increased long-term social and economic costs.
